# A nomogram for predicting free flap necrosis in soft tissue reconstruction of lower limbs: a retrospective cohort study

**DOI:** 10.3389/fmed.2026.1751658

**Published:** 2026-05-26

**Authors:** Cong Cheng, Xiaoyu Huang, Hai Liang, Zongyuan Jiang

**Affiliations:** Department of Hand Surgery, People’s Hospital of Longhua, Shenzhen, China

**Keywords:** flap necrosis, free flap, nomogram, predictive model, soft tissue defects

## Abstract

**Objective:**

To identify independent risk factors for free flap necrosis in lower limb soft tissue reconstruction and to develop and internally validate a preliminary nomogram for risk prediction.

**Methods:**

A retrospective cohort study was conducted on patients who underwent free flap reconstruction for lower limb soft tissue defects between January 2010 and March 2025. Eligible patients were randomly split into a training cohort (70%) for model development and a validation cohort (30%) for internal validation. Variable selection were performed solely using Least Absolute Shrinkage and Selection Operator (LASSO) regression. A nomogram was constructed based on the identified risk factors, and its performance was rigorously evaluated via bootstrap internal validation (500 repetitions with optimism correction) across three key dimensions: discrimination (optimism-corrected area under the receiver operating characteristic curve, AUC), calibration (calibration slope/intercept and Brier score), and clinical utility (decision curve analysis, DCA).

**Results:**

A total of 220 patients were enrolled in this study. Of these, 154 patients were assigned to the training cohort and 66 patients to the validation cohort. Five independent risk factors were identified: Gustilo-Anderson classification IIIB/IIIC (OR = 3.74, 95% CI: 1.89–7.41), preoperative D-dimer > 0.5 mg/L (OR = 3.16, 95% CI: 1.50–6.64), preoperative albumin < 35 g/L (OR = 2.75, 95% CI: 1.41–5.34), operative time > 6 h (OR = 2.59, 95% CI: 1.35–4.94), and defect size > 50 cm^2^ (OR = 2.05, 95% CI: 1.10–3.83). The nomogram showed promising discriminative ability. The optimism-corrected AUC was 0.87 (95% CI: 0.84–0.90) in the training cohort with excellent calibration (slope = 1.00, intercept = 0.00) and a Brier score of 0.122. In the validation cohort, the AUC was 0.86 (95% CI: 0.81–0.91) with a Brier score of 0.130. The DCA demonstrated that the nomogram had superior net clinical benefit compared to “treat all” or “treat none” strategies.

**Conclusion:**

Our study developed and internally validated a preliminary nomogram incorporating five preoperative factors for predicting free flap necrosis in lower limb soft tissue reconstruction. The model demonstrated robust discrimination, excellent calibration, and meaningful clinical utility in the single-center cohort. However, its generalizability and real-world clinical utility requires confirmation through prospective external validation in multi-center settings.

## Introduction

Lower limb soft tissue defects, predominantly caused by high-energy trauma tumor resection, and chronic ulcers, are a major clinical challenge in orthopedics and microsurgery. Epidemiological data indicated that such defect cases account for 35% of all extremity trauma cases with Gustilo-Anderson IIIB/IIIC fractures, contributing to over 60% of severe soft tissue loss (1). The reconstruction of these defects is not merely cosmetic. It also directly impacts limb salvage, functional recovery, and quality of life, as delayed or inadequate repair can lead to amputation in 12–28% of cases and lifelong disability in nearly half of patients (2).

For such defects, free flap reconstruction has become the gold standard of treatment due to its ability to provide sufficient tissue volume, reliable blood supply and functional recovery. Free flap techniques have revolutionized lower limb reconstruction over the past three decades. They enhanced the rate of limb salvage from 65% to over 90% in severe trauma cases (3, 4). However, free flap necrosis, which is defined as partial or total loss of the transferred tissue due to vascular compromise, remains a devastating complication that occurs in 8–15% of cases (5). Besides the direct loss of the flap, the necrosis often leads to prolonged hospital stays, increases healthcare costs, and raises risks of secondary infections and amputation (1, 6).

Numerous studies have attempted to identify risk factors for free flap necrosis in lower limb reconstruction, yet critical gaps persist in the current evidence base. Early research primarily focused on single procedural factors. Godina et al. emphasized the role of delayed reconstruction > 72 h in increasing complication rates (7), while Gürlek et al. linked prolonged ischemic time to endothelial damage (8). More recent studies have expanded to include systemic factors, such as diabetes mellitus and hypoalbuminemia. However, these investigations often suffered from limitations in small sample sizes, single-center biases, and fragmented analysis of risk factors (9–11).

Existing literature lacks a comprehensive integration of systemic, local, and procedural factors to identify independent predictors of flap necrosis. Schuderer et al. (12) highlighted the impact of surgical site infection on flap survival, but few studies have quantified its interaction with hypercoagulable states or nutritional status, which may synergistically increase necrosis risk. A large multicenter analysis of 4,675 trauma patients identified that prereconstruction vascular interventions were strongly associated with increased rates of surgical site infection and amputation, while free flap reconstruction was correlated with prolonged hospital stay. However, it did not provide a targeted predictive tool for free flap necrosis (13). Additionally, risk prediction models for free flap outcomes in lower limbs remain scarce. Most available models focus on head and neck or breast reconstruction, and their applicability to lower limb trauma is limited by differences in wound biology and patient demographics (4, 5).

The Gustilo-Anderson classification, which is regarded as a cornerstone of open fracture management, further underscores the need for targeted risk stratification. Patients with Gustilo-Anderson IIIB/IIIC fractures that were characterized by extensive soft tissue loss and arterial injury, had drastically higher complication rates than those with milder injuries (14). However, current guidelines do not specify how to tailor perioperative management based on the interplay between fracture severity and other systemic risk factors (15–17). This gap made clinicians reliant on empirical judgment rather than evidence-based strategies to mitigate necrosis risk.

Against this backdrop, our retrospective study aimed to identify independent risk factors for free flap necrosis in a large cohort of patients with lower limb soft tissue defects, quantify the relative contribution of each risk factor by using Least Absolute Shrinkage and Selection Operator (LASSO) regression, and provide clinically actionable insights to optimize perioperative management by developing a nomogram. We hypothesized that this nomogram would be a reliable tool for risk stratification, and it would help clinicians identify high-risk patients and implement targeted interventions to reduce free flap necrosis rates.

## Materials and methods

### Study design

This retrospective cohort study enrolled patients who underwent free flap reconstruction for lower limb soft tissue defects at our institution between January 2010 and March 2025. The study protocol was approved by the Institutional Review Board of People’s Hospital of Longhua, Shenzhen and adheres to the ethical principles of the Declaration of Helsinki. Informed consent was waived due to the retrospective nature of the study, with all patient data de-identified to protect confidentiality.

Patients were included if they met the following criteria: (1) they had unilateral lower limb soft tissue defects and underwent primary free flap reconstruction; (2) they had complete clinical, laboratory, and surgical records, as well as ≥ 3 months of postoperative follow-up conducted by in-person visits or telephone interviews; (3) free flap necrosis was confirmed by both clinical examination, which was manifested as flap discoloration, loss of capillary refill, or absence of Doppler signals. Free flap necrosis was defined as partial necrosis (necrosis involving ≤ 60% of the flap area, requiring surgical debridement or secondary repair) or total necrosis (necrosis involving > 60% of the flap area, requiring complete flap removal or reoperation), which was consistent with Clavien-Dindo grade II-V complications (14). Patients were excluded as followings: (1) they underwent secondary flap surgery, revision vascular anastomosis, or flap salvage procedures; (2) necrosis was attributed to technical errors documented in operative reports, including pedicle kinking/torsion, anastomotic stenosis requiring revision, flap inset-related compression, intimal flap or suture rupture, or iatrogenic pedicle injury; (3) they had comorbidities that precluded flap viability, such as end-stage peripheral vascular disease, severe immunodeficiency, or malignant tumors with distant metastasis; (4) they had missing key variables or were lost to follow-up; (5) they had a history of lower limb radiotherapy with a total dose ≥ 60 Gy and unresolved tissue fibrosis. All operative notes of patients with flap necrosis were independently reviewed by two senior microsurgeons who were blinded to the patients’ flap necrosis status and the study’s objectives. The review was conducted prior to the LASSO analysis, and the reviewers were unaware of the final model variables. Cases were classified as technical error only when both reviewers agreed on the presence of at least one of the above criteria. Disagreements were resolved by consensus with a third reviewer.

### Data collection

Clinical data were extracted from electronic medical records and a prospectively maintained microsurgery database by two independent researchers. Discrepancies in data extraction were resolved through arbitration by a third researcher with expertise in microsurgical outcomes. A 10% random sample of records was cross-validated to ensure data accuracy. For the purpose of developing a preoperative prediction model, only preoperative and intraoperative variables available before or during surgery were included as candidate predictors in the model. Postoperative outcomes, such as surgical site infection, were excluded. Variables collected included demographic and clinical characteristics, including age, sex, body mass index (BMI, categorized as < 18.5, 18.5–24.9, and ≥ 25 kg/m^2^), American Society of Anesthesiology (ASA) physical status classification, smoking history (never, former, and current), alcohol consumption, cardiovascular disease (coronary heart disease or peripheral vascular disease), diabetes mellitus (diagnosed via HbA1c ≥ 6.5% or medication use), and hypertension (diagnosed via blood pressure ≥ 140/90 mmHg or medication use). Preoperative factors included surgeon experience (< 5 years and ≥ 5 years), Gustilo-Anderson classification (II/IIIA and IIIB/IIIC), soft tissue defect site (proximal leg, mid-leg, distal leg, and foot-ankle), defect size (> 50 cm^2^and ≦50 cm^2^), time from injury to surgery (< 72, 72 h to 7 days, and > 7 days), history of preoperative radiotherapy (dose ≥ 60 Gy), preoperative D-dimer level (≤ 0.5 and > 0.5 mg/L), preoperative hemoglobin (<120 and ≥ 120 g/L) and albumin (< 35 and ≥ 35 g/L) levels. Intraoperative factors included free flap type, flap size (≤ 40 and > 40 cm^2^), blood loss (≤ 500 and > 500 mL), operative time (≤ 6 and > 6 h), intraoperative blood transfusion (yes or no). Patients were randomly assigned to a training cohort (70%) for model development and an a validation cohort (30%) for internal validation.

Prior to analysis, we assessed the extent and pattern of missing data across all candidate variables. Missingness was limited (<5% of all data points) and assumed to be missing at random (MAR). The specific variables with missing values, along with their missingness proportions, were as follows: preoperative albumin (2.3%, *n* = 5), preoperative D-dimer (1.8%, *n* = 4), and preoperative hemoglobin (1.4%, *n* = 3). No other variables had missing data. We employed multiple imputation by chained equations (MICE) to handle these missing values, as it preserves the variability and uncertainty associated with imputation. By using the mice package in R, we generated 10 imputed datasets. The imputation model included all candidate predictors and outcome variables to ensure appropriate conditional relationships. The imputation process ran for 20 iterations to achieve convergence. The LASSO regression model was then developed separately on each of the 10 imputed datasets. The regression coefficients and performance metrics from each model were pooled according to Rubin’s rules to obtain final estimates and confidence intervals that account for the uncertainty due to missing data. Median imputation was not used for any variable to avoid introducing bias and underestimating variance.

### Model development and internal validation

To enhance model robustness and mitigate overfitting given the limited number of events, we employed a single-stage penalized regression approach for variable selection and model construction. Specifically, the LASSO regression was applied to all candidate preoperative and intraoperative variables that were deemed clinically relevant. The LASSO regression simultaneously performed variable selection and coefficient shrinkage by imposing an L1 penalty, which was particularly suitable for datasets with a relatively high number of predictors compared to events. The optimal tuning parameter (λ) was determined by 10-fold cross-validation to select the value that minimized the binomial deviance. The variables with non-zero coefficients at this optimal λ were retained to form the final prediction model.

The performance of the final LASSO model was rigorously evaluated through bootstrap internal validation with optimism correction. We performed 500 bootstrap resamples from the training cohort. Within each resample, the entire modeling process including the LASSO penalty parameter selection by cross-validation was repeated. The model was then applied to the bootstrap sample to obtain the apparent performance, and to the original training cohort to obtain the test performance.

### Model performance assessment

A nomogram was constructed based on the regression coefficients of independent risk factors, and each variable was assigned a weighted score. The total score of each patient was mapped to the predicted probability of flap necrosis. Model performance was assessed in terms of discrimination, calibration, and overall accuracy. Discrimination, defined as the ability to differentiate between patients with and without flap necrosis, was quantified by the area under the receiver operating characteristic curve (AUC), both as the apparent AUC and the bootstrap optimism-corrected AUC. Calibration, which reflected the agreement between predicted probabilities and observed outcomes, was evaluated using calibration plots. We reported the calibration slope and intercept from the calibration plot, where an ideal model had a slope of 1 and an intercept of 0. The Brier score, calculated as the mean squared difference between predicted probabilities and actual outcomes, was used to assess overall predictive accuracy, and lower scores indicated better accuracy. Clinical utility was evaluated using decision curve analysis (DCA) to compare the net benefit of the nomogram against “treat all” and “treat none” strategies across a range of threshold probabilities.

### Statistical analysis

Statistical analyses were performed by SPSS 25.0 (IBM Corporation, United States) and R version 4.2.2 (R Foundation for Statistical Computing, Vienna, Austria). Continuous variables were described as mean ± standard deviation or median (interquartile range) based on their distribution, and categorical variables as frequencies (percentages). The normality of continuous variables was assessed by the Shapiro-Wilk test. Comparisons between patients with and without flap necrosis were performed using independent samples *t*-tests or Mann-Whitney U tests for continuous variables, and chi-square tests or Fisher’s exact tests for categorical variables. A two-tailed *p* < 0.05 was considered statistically significant for descriptive comparisons. No *p*-value threshold was applied for variable entry into the LASSO regression model.

## Results

### Baseline characteristics of patients

**FIGURE 1:**
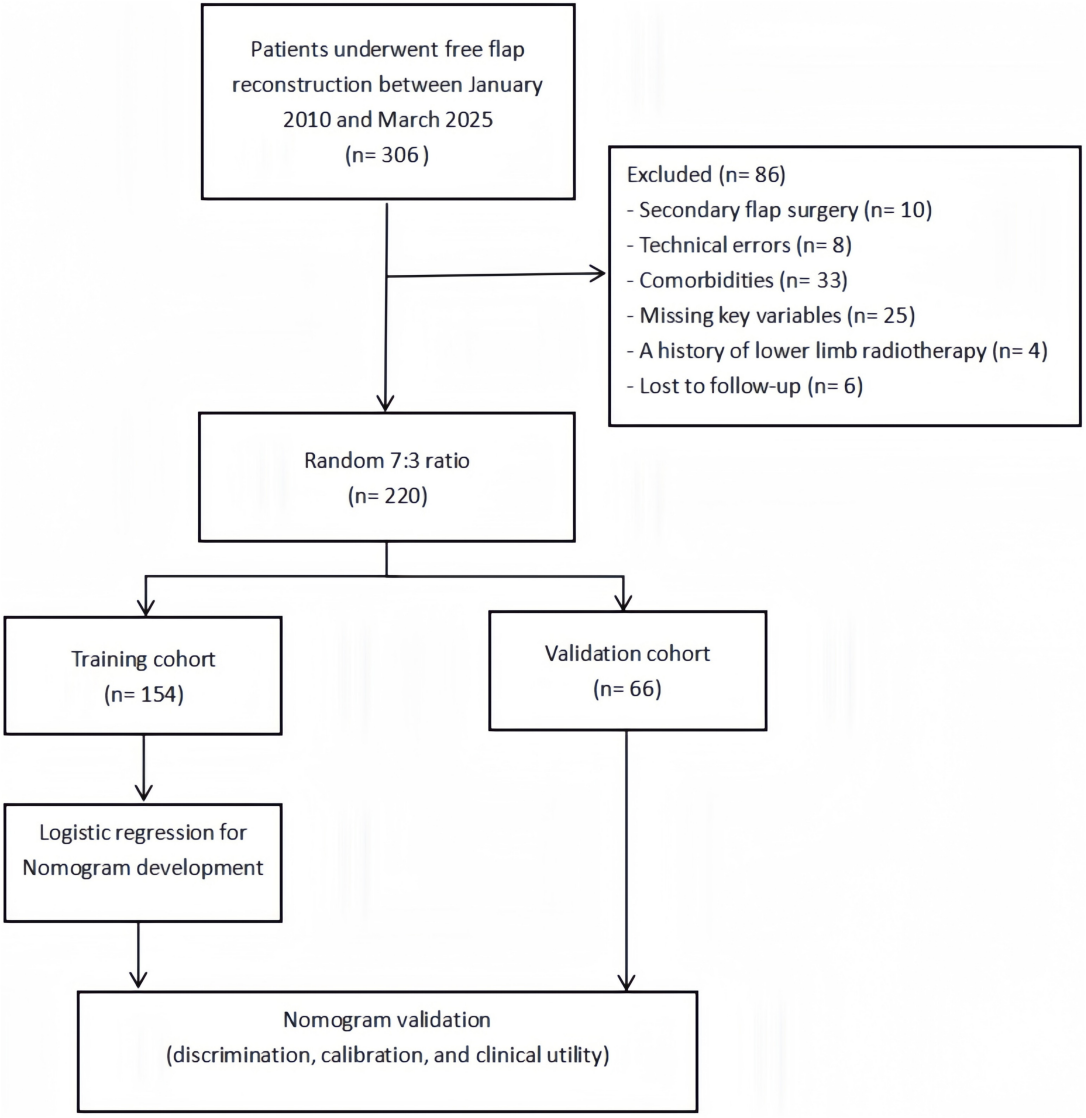
Patient screening flowchart.

A total of 306 patients who underwent free flap reconstruction for lower limb soft tissue defects at our institution between January 2010 and March 2025 were initially screened. After excluding 86 patients, 220 eligible patients were finally enrolled in this retrospective cohort study (Figure 1). All patients completed a follow-up of at least 3 months, and were randomly assigned to the training cohort (*n* = 154) and validation cohort (*n* = 66). Among them, 44 patients (20.0%) developed free flap necrosis, including 18 cases of partial necrosis and 26 cases of total necrosis that required complete flap removal or reoperation. The remaining 176 patients (80.0%) had no flap necrosis and achieved satisfactory wound healing.

The demographic and clinical characteristics are summarized in Table 1. Baseline characteristic comparison showed no significant differences in all variables between the training and validation cohorts (all *P* > 0.05), confirming good comparability. In the total cohort, most patients were male (162 cases, 73.6%), with age distribution concentrated in the 30–50 years group (98 cases, 44.5%). In terms of comorbidities, 35 patients (15.9%) had diabetes mellitus, 32 patients (14.5%) had cardiovascular disease, and 77 patients (35.0%) were current smokers. From the perspective of surgical-related factors, 92 patients (41.8%) had Gustilo-Anderson classification IIIB/IIIC injuries, 90 patients (40.9%) had soft tissue defects larger than 50 cm^2^.

**TABLE 1:** The demographic and clinical characteristics of patients in total cohort and subgroup analysis.

Variable	Total (*n* = 220)	Necrosis (*n* = 44)	No necrosis (*n* = 176)	*P*-value	Training set (*n* = 154)	Validation set (*n* = 66)	*P*-value
Sex							0.912
Male	162 (73.6%)	33 (75.0%)	129 (73.3%)	0.845	113 (73.4%)	49 (74.2%)	
Female	58 (26.4%)	11 (25.0%)	47 (26.7%)		41 (26.6%)	17 (25.8%)	
Age (years)							0.876
<30	75 (34.1%)	9 (20.5%)	66 (37.5%)	0.029	53 (34.4%)	22 (33.3%)	
30–50	98 (44.5%)	23 (52.3%)	75 (42.6%)		69 (44.8%)	29 (43.9%)	
>50	47 (21.4%)	12 (27.3%)	35 (19.9%)		32 (20.8%)	15 (22.8%)	
BMI (kg/m^2^)							0.935
<18.5	26 (11.8%)	9 (20.5%)	17 (9.7%)	0.063	18 (11.7%)	8 (12.1%)	
18.5 24.9	145 (65.9%)	26 (59.1%)	119 (67.6%)		102 (66.2%)	43 (65.2%)	
≥25	49 (22.3%)	9 (20.5%)	40 (22.7%)		34 (22.1%)	15 (22.7%)	
ASA classification							0.821
I-II	159 (72.3%)	26 (59.1%)	133 (75.6%)	0.031	111 (72.1%)	48 (72.7%)	
III-IV	61 (27.7%)	18 (40.9%)	43 (24.4%)		43 (27.9%)	18 (27.3%)	
Smoking history							0.795
Never	105 (47.7%)	16 (36.4%)	89 (50.6%)	0.025	74 (48.1%)	31 (47.0%)	
Former	38 (17.3%)	7 (15.9%)	31 (17.6%)		27 (17.5%)	11 (16.7%)	
Current	77 (35.0%)	21 (47.7%)	56 (31.8%)		53 (34.4%)	24 (36.4%)	
Alcohol consumption							0.864
No	142 (64.5%)	26 (59.1%)	116 (65.9%)	0.412	99 (64.3%)	43 (65.2%)	
Yes	78 (35.5%)	18 (40.9%)	60 (34.1%)		55 (35.7%)	23 (34.8%)	
Cardiovascular disease							0.907
No	188 (85.5%)	34 (77.3%)	154 (87.5%)	0.089	132 (85.7%)	56 (84.8%)	
Yes	32 (14.5%)	10 (22.7%)	22 (12.5%)		22 (14.3%)	10 (15.2%)	
Diabetes mellitus							0.832
No	185 (84.1%)	32 (72.7%)	153 (86.9%)	0.042	130 (84.4%)	55 (83.3%)	
Yes	35 (15.9%)	12 (27.3%)	23 (13.1%)		24 (15.6%)	11 (16.7%)	
Hypertension							0.751
No	160 (72.7%)	30 (68.2%)	130 (73.9%)	0.485	112 (72.7%)	48 (72.7%)	
Yes	60 (27.3%)	14 (31.8%)	46 (26.1%)		42 (27.3%)	18 (27.3%)	
Surgeon experience							0.9
< 5 Years	99 (45.0%)	21 (47.7%)	78 (44.3%)	0.69	69 (44.8%)	30 (45.5%)	
≥ 5 Years	121 (55.0%)	23 (52.3%)	98 (55.7%)		85 (55.2%)	36 (54.5%)	
Gustilo- Anderson classification							0.943
II/IIIA	128 (58.2%)	15 (34.1%)	113 (64.2%)	<0.001	90 (58.4%)	38 (57.6%)	
IIIB/IIIC	92 (41.8%)	29 (65.9%)	63 (35.8%)		64 (41.6%)	28 (42.4%)	
Soft tissue defect site							0.918
Proximal leg	52 (23.6%)	7 (15.9%)	45 (25.6%)	0.038	37 (24.0%)	15 (22.7%)	
Mid leg	68 (30.9%)	13 (29.5%)	55 (31.2%)		48 (31.2%)	20 (30.3%)	
Distal leg	55 (25.0%)	14 (31.8%)	41 (23.3%)		39 (25.3%)	16 (24.2%)	
Foot-ankle	45 (20.5%)	10 (22.7%)	35 (19.9%)		30 (19.5%)	15 (22.7%)	
Defect size (cm^2^)							0.896
≤.8	130 (59.1%)	17 (38.6%)	113 (64.2%)	0.003	91 (59.1%)	39 (59.1%)	
>50	90 (40.9%)	27 (61.4%)	63 (35.8%)		63 (40.9%)	27 (40.9%)	
Time from injury to surgery							0.925
<72 h	80 (36.4%)	8 (18.2%)	72 (40.9%)	0.008	56 (36.4%)	24 (36.4%)	
72 h–7 days	95 (43.2%)	26 (59.1%)	69 (39.2%)		67 (43.5%)	28 (42.4%)	
>7 days	45 (20.4%)	10 (22.7%)	35 (19.9%)		31 (20.1%)	14 (21.2%)	
Preoperative radiotherapy (dose ≥60 Gy)							0.871
No	195 (88.6%)	36 (81.8%)	159 (90.3%)	0.045	136 (88.3%)	59 (89.4%)	
Yes	25 (11.4%)	8 (18.2%)	17 (9.7%)		18 (11.7%)	7 (10.6%)	
Preoperative D-dimer (mg/L)							0.901
≤.90	120 (54.5%)	14 (31.8%)	106 (60.2%)	0.001	85 (55.2%)	35 (53.0%)	
>0.5	100 (45.5%)	30 (68.2%)	70 (39.8%)		69 (44.8%)	31 (47.0%)	
Preoperative hemoglobin (g/L)							0.867
≥.86	135 (61.4%)	18 (40.9%)	117 (66.5%)	0.004	95 (61.7%)	40 (60.6%)	
<120	85 (38.6%)	26 (59.1%)	59 (33.5%)		59 (38.3%)	26 (39.4%)	
Preoperative albumin (g/L)							0.932
≥.9	140 (63.6%)	19 (43.2%)	121 (68.8%)	0.002	98 (63.6%)	42 (63.6%)	
<35	80 (36.4%)	25 (56.8%)	55 (31.2%)		56 (36.4%)	24 (36.4%)	
Free flap type							0.978
ATF	90 (40.9%)	18 (40.9%)	72 (40.9%)	0.987	63 (40.9%)	27 (40.9%)	
LDMF	50 (22.7%)	11 (25.0%)	39 (22.2%)		35 (22.7%)	15 (22.7%)	
DIEP	40 (18.2%)	8 (18.2%)	32 (18.2%)		28 (18.2%)	12 (18.2%)	
Others	40 (18.2%)	7 (15.9%)	33 (18.8%)		28 (18.2%)	12 (18.2%)	
Flap size (cm^2^)							0.889
≤.8	125 (56.8%)	15 (34.1%)	110 (62.5%)	<0.001	88 (57.1%)	37 (56.1%)	
>40	95 (43.2%)	29 (65.9%)	66 (37.5%)		66 (42.9%)	29 (43.9%)	
Blood loss (mL)							0.913
≤.91	140 (63.6%)	20 (45.5%)	120 (68.2%)	0.012	98 (63.6%)	42 (63.6%)	
>500	80 (36.4%)	24 (54.5%)	56 (31.8%)		56 (36.4%)	24 (36.4%)	
Operative time (h)							0.879
≤.	135 (61.4%)	16 (36.4%)	119 (67.6%)	<0.001	94 (61.0%)	41 (62.1%)	
>6	85 (38.6%)	28 (63.6%)	57 (32.4%)		60 (39.0%)	25 (37.9%)	
Intraoperative blood transfusion							0.846
No	160 (72.7%)	26 (59.1%)	134 (76.1%)	0.039	112 (72.7%)	48 (72.7%)	
Yes	60 (27.3%)	18 (40.9%)	42 (23.9%)		42 (27.3%)	18 (27.3%)	

### Analysis of risk factors

**FIGURE 2:**
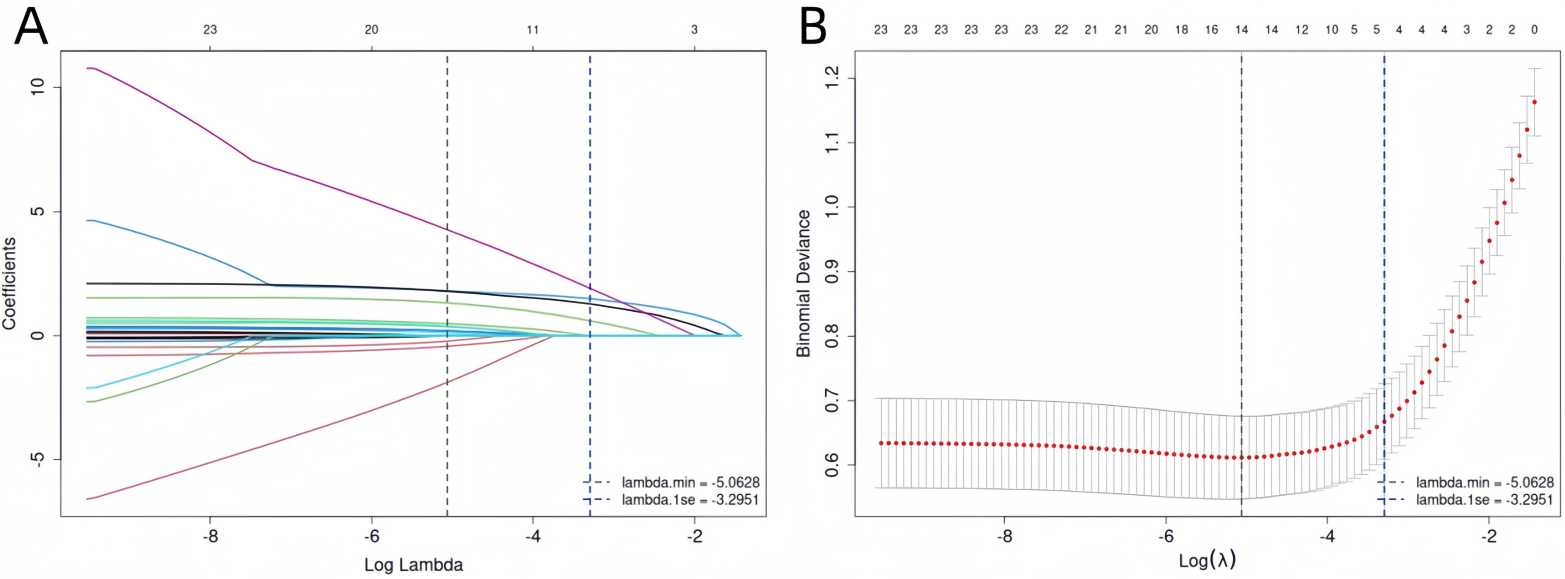
LASSO regression analysis for free flap necrosis in lower limb soft tissue reconstruction. **(A)** The LASSO regression coefficient trajectory plot (changes with log λ, ultimately screening 5 risk factors with non-zero coefficients). **(B)** The cross-validation plot (the optimal λ value was determined by the minimum partial likelihood deviance for risk factor screening and dimensionality reduction).

A total of 23 candidate preoperative and intraoperative variables were entered into the LASSO regression analysis for variable selection. The trajectory of LASSO coefficients as the penalty parameter (λ) varied is shown in Figure 2A. By using 10-fold cross-validation, the optimal λ value was selected, corresponding to the minimum binomial deviance (Figure 2B). At this optimal λ, the LASSO model retained 5 independent predictors with non-zero coefficients: Gustilo-Anderson classification IIIB/IIIC, preoperative D-dimer > 0.5 mg/L, operative time > 6 h, preoperative albumin < 35 g/L, and defect size > 50 cm^2^. The coefficients (log-odds), standard errors, and odds ratios for the final LASSO model are presented in Table 2.

**TABLE 2:** Independent predictors retained in the final LASSO regression model for predicting flap necrosis.

Variable	Category (vs. reference)	Coefficient (β)	Standard error (SE) *	Odds ratio (OR)	95% CI for OR	*P*-value
Gustilo-Anderson classification	IIIB/IIIC (vs. I/II/IIA)	1.32	0.35	3.74	(1.89, 7.41)	<0.001
Preoperative D-dimer	>0.5 mg/L (vs. ≤0.5 mg/L)	1.15	0.38	3.16	(1.50, 6.64)	0.003
Preoperative albumin	<35 g/L (vs. ≥35 g/L)	1.01	0.34	2.75	(1.41, 5.34)	0.003
Operative time	>6 h (vs. ≤6 h)	0.95	0.33	2.59	(1.35, 4.94)	0.004
Defect size	>50 cm^2^ (vs. ≤50 cm^2^)	0.72	0.32	2.05	(1.10, 3.83)	0.024

*Standard error of the coefficient (β).

To evaluate the combined effect of elevated D-dimer (> 0.5 mg/L) and hypoalbuminemia (< 35 g/L) on flap necrosis risk, we first performed a descriptive 2 × 2 subgroup analysis based on the entire cohort (*n* = 220). Among the 47 patients with both risk factors (double-positive group), 29 developed flap necrosis, yielding a necrosis rate of 61.7% (29/47). In contrast, among the 92 patients with neither risk factor (double-negative group), only 8 developed necrosis, corresponding to a rate of 8.7% (8/92). Patients with isolated elevated D-dimer (*n* = 38) had a necrosis rate of 31.6% (12/38), and those with isolated hypoalbuminemia (*n* = 43) had a rate of 27.9% (12/43).

To formally test the synergistic effect between elevated D-dimer and hypoalbuminemia, we performed a *post-hoc* exploratory analysis by adding a product term (D-dimer × albumin) to the multivariable logistic regression model that already included the five independent predictors. The interaction term was statistically significant (OR = 2.87, 95% CI: 1.35nteract*P* = 0.006), indicating a multiplicative synergistic effect beyond the independent contributions of each factor. The area under the ROC curve of the model with the interaction term was 0.89 (95% CI: 0.86–0.92), comparable to the original model without interaction (0.88).

These findings supported the hypothesis that concurrent hypercoagulability (elevated D-dimer) and poor nutritional status (hypoalbuminemia) exerted a “double-hit” synergistic effect on free flap necrosis in lower limb reconstruction.

### Development and validation of the nomogram

**FIGURE 3:**
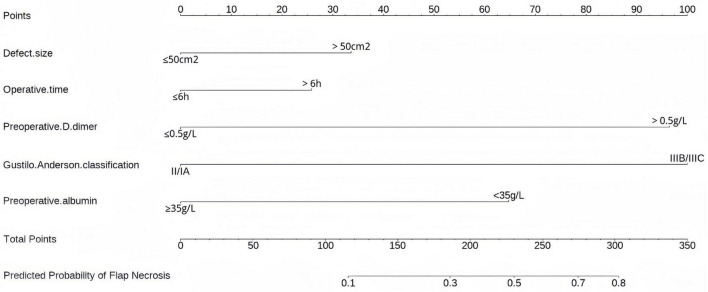
Nomogram for predicting the risk of free flap necrosis in lower limb soft tissue defect reconstruction. The nomogram includes the score scales of 5 independent risk factors, and the predicted probability range (10–80%) of free flap necrosis corresponding to the total score.

A nomogram for predicting the risk of free flap necrosis in lower limb soft tissue reconstruction was constructed based on the regression coefficients of the 5 independent risk factors (Figure 3). Each risk factor was assigned a weighted score according to its degree of influence. For individual patients, the total score was calculated by summing the scores of each risk factor, and the corresponding predicted probability of flap necrosis (ranging from 10 to 80%) could be obtained by mapping the total score to the nomogram.

**FIGURE 4:**
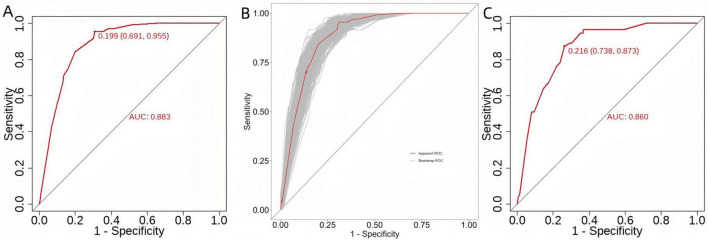
ROC curves of the nomogram for predicting free flap necrosis in lower limb soft tissue defect reconstruction. **(A)** Apparent AUC in the training cohort. **(B)** Optimism-corrected AUC in the training cohort. **(C)** AUC in the validation cohort.

The discriminative ability of the nomogram in the training cohort, as measured by the apparent AUC, was 0.88 (95% CI: 0.85, 0.91) (Figure 4A). After applying bootstrap optimism correction (500 repetitions), the corrected AUC was 0.87 (95% CI: 0.84, 0.90) (Figure 4B). In the internal validation cohort, the AUC was 0.86 (95% CI: 0.81, 0.91) (Figure 4C). The ROC curves for both cohorts are shown in Figure 4.

**FIGURE 5:**
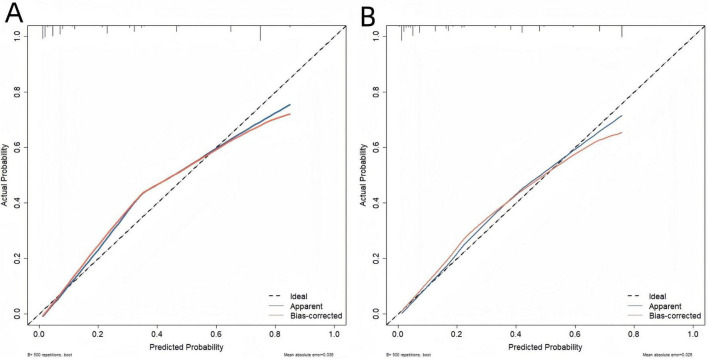
Calibration curves of the nomogram for predicting free flap necrosis in lower limb soft tissue defect reconstruction. **(A)** The training cohort. **(B)** The validation cohort. Both include the ideal curve (perfect consistency between predicted and actual outcomes), apparent curve (uncorrected), and Bootstrap-corrected curve (*B* = 500 repetitions), indicating good consistency between predicted probability and actual necrosis risk.

Calibration plots demonstrated good agreement between predicted probabilities and observed outcomes (Figure 5). The calibration slope was 1.00 (95% CI: 0.82, 1.18) and the calibration intercept was 0.00 (95% CI: −0.26, 0.26) in the optimism-corrected training cohort with a mean absolute error (MAE) of 0.036, which showed minimal overfitting and good calibration. In the validation cohort, the calibration slope was 0.91 (95% CI: 0.65, 1.17), intercept was 0.02 (95%CI: −0.37, 0.41), and MAE was 0.026. The Brier score, that reflected overall prediction error, was 0.122 (95%CI: 0.105, 0.140) in the training cohort and 0.130 (95%CI: 0.102, 0.158) in the validation cohort. Thus the model achieved good overall predictive accuracy across both cohorts, and the minimal difference further supports the model’s robustness.

**FIGURE 6:**
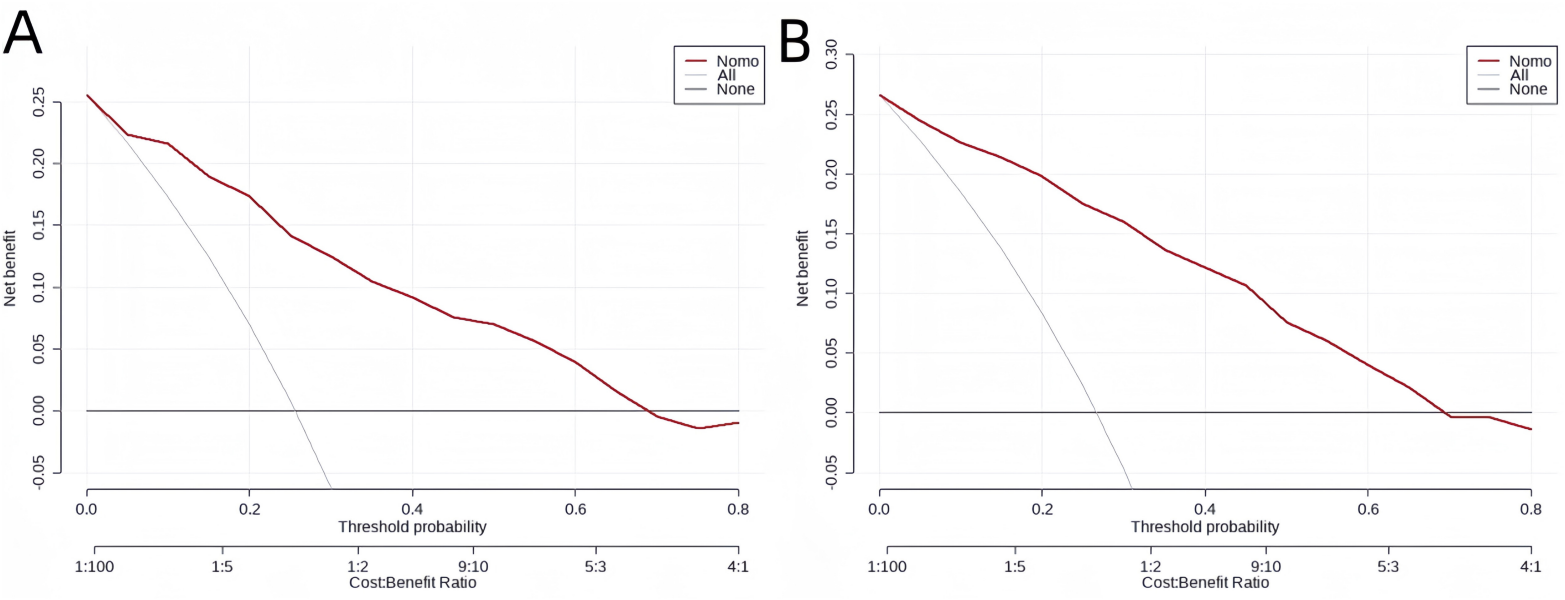
Decision curve analysis of the nomogram for predicting free flap necrosis in lower limb soft tissue defect reconstruction. **(A)** The training cohort. **(B)** The validation cohort. It compares the net benefit of the nomogram (mo_model) with the “treat all” and “treat none” strategies.

Decision curve analysis (DCA) demonstrated the nomogram to guide clinical decisions could yield significant net benefits compared to the strategies of “treating all patients” as high-risk or “treating none.” These benefits were observed within a risk threshold probability range of 1–70% in the training cohort and 3–68% in the validation cohort (Figure 6).

## Discussion

Our study enrolled 220 patients who underwent free flap reconstruction for lower limb soft tissue defects, and identified 5 independent risk factors for free flap necrosis by LASSO regression: Gustilo-Anderson classification IIIB/IIIC, preoperative D-dimer > 0.5 mg/L, operative time > 6 h, preoperative albumin < 35 g/L, and defect size > 50 cm^2^, which ultimately constructed a clinically applicable nomogram. The results not only confirmed the importance of classic risk factors in flap survival, but also provided a quantitative tool for preoperative risk prediction.

Our findings revealed that the Gustilo-Anderson classification IIIB/IIIC was the most significant risk factor in our study with a necrosis rate of 65.9% in this subgroup, nearly double that of II/IIIA (34.1%). Olesen et al. (15) specifically noted that IIIB/IIIC fracture had a 31.5% flap necrosis rate when compared to 12.8% for IIIA fractures due to compromised recipient site vascular reserve and increased bacterial colonization. Perrot et al. (18) reported that lower limb defects from high-energy trauma had a 2.9-fold higher flap failure rate than low-energy injuries, as the zone of injury extended beyond visible tissue damage to impair microcirculation. Our study linked the Gustilo-Anderson classification directly to free flap outcomes. Early microsurgical reconstruction (<72 h) of IIIB/IIIC fractures with aggressive debridement of nonviable tissue could reduce necrosis risk by 40% (7). It underscored the importance of integrating the Gustilo-Anderson classification into preoperative planning, such as prioritizing urgent flap coverage and multimodal infection prophylaxis in IIIB/IIIC cases.

Preoperative D-dimer > 0.5 mg/L was present in 68.2% of patients who developed flap necrosis. Bui et al. (19) evaluated 1,193 free flaps, and found that hypercoagulability, indicated by elevated D-dimer or fibrinogen, was responsible for 38% of venous thrombosis cases, which was the leading cause of acute flap failure. A meta-analysis of lower limb free flaps documented that D-dimer > 0.5 mg/L was associated with a 2.7-fold higher risk of anastomotic thrombosis, as increased fibrin deposition occluded small-caliber vessels in the flaps (20). Persson et al. (21) proposed that perioperative red blood cell transfusion was common in trauma patients with elevated D-dimer, and it increased flap compromise risk by altering immune-mediated coagulation regulation. They also suggested that D-dimer may not only predict risk, but also identify patients who would benefit from perioperative anticoagulation to preserve flap patency.

Our study demonstrated that preoperative albumin < 35 g/L increased flap necrosis risk by 2.75-fold. Offodile et al. (10) reported that hypoalbuminemia < 3.0 g/dL was the strongest predictor of prolonged hospital stay and wound complications in free flap patients. Albumin supplementation should be initiated at least 72 h preoperatively to normalize levels. Ouyang et al. (22) similarly observed that albumin < 35 g/L raised risk of flap necrosis by 4.47-fold in oral and maxillofacial reconstruction, and albumin could maintain colloid osmotic pressure to reduce tissue edema and support fibroblast proliferation for anastomotic healing. Thus preoperative nutritional screening and targeted supplementation were essential to improve flap outcomes in at-risk patients (23).

Defect size > 50 cm^2^ was another important independent predictor in our study. Shasti et al. (24) found that soft-tissue defects > 200 cm^2^ had a 100% complication rate in acute trauma, and even moderate-sized defect > 50 cm^2^ had a 2.3-fold higher risk of partial/total flap failure. The underlying mechanism lied in the zone of injury expansion. Large defects often involved more extensive damage to surrounding blood vessels and soft tissue, ultimately reducing the recipient sitearge defects often involved more extensive damage ficients of the 5 indepe(19). Moreover, large defects required large flaps with higher metabolic demand that prolonged cold ischemia time, as each additional 30 min of ischemia increased ischemia reperfusion injury (IRI)-induced endothelial damage by 15% (8).

Operative time > 6 h synergistically exacerbated this risk, as prolonged surgery increased flap exposure to ischemia and hemodynamic instability (10). Collins et al. (25) noted that operative time > 6 h was correlated to a 5.65-fold higher risk of venous thrombosis, as prolonged anesthesia-induced vasoconstriction reduced flap perfusion. Xiong et al. (20) declared that the combination of large defect size > 50 cm^2^ and operative time > 6 h raised necrosis risk by 4.1-fold, as the dual burden of ischemia and tissue trauma overwhelmed the flap’s reparative capacity. The optimized surgical planning was required to mitigate IRI and endothelial damage, such as using perforator flaps to minimize flap size or dual-surgeon teams to reduce operative time (26).

Our study found no significant association between diabetes and free flap necrosis. Kantar et al. (9) explained that chronic hyperglycemia induced glycation of endothelial proteins, thereby reducing nitric oxide availability and increasing vascular permeability, both of which impaired flap microcirculation. Iamaguchi et al. (27) observed that diabetic patients with poor glycemic control (HbA1c > 7%) had a 5.4-fold higher complication rate in lower limb free flaps, and hyperglycemia suppressed immune function and delayed wound healing as well. It was also suggested that perioperative glycemic control was more critical than antibiotic escalation for diabetic patients (9).

Our study built on existing literature by integrating multiple risk factors into a single predictive model. Reece et al. (28) assessed 91 fasciocutaneous flaps, but did not include systemic factors like D-dimer. While Shasti et al. (24) focused on defect size > 200 cm^2^ as a risk factor, our study identified a smaller cutoff for necrosis. Levin et al. (13) highlighted the critical impact of severe injury patterns and vascular compromise on reconstruction outcomes, and they emphasized the importance of injury severity stratification. Our study corroborates this by identifying Gustilo-Anderson IIIB/IIIC classification as the strongest predictor in our nomogram. Another novel finding of our study was the interaction between D-dimer and albumin. Patients with both D-dimer > 0.5 mg/L and albumin < 35 g/L had a 62.3% necrosis rate when compared to 8.7% for patients with neither factor. This synergistic effect had not been previously reported but was biologically plausible. Hypoalbuminemia exacerbated edema-induced vascular compression, while hypercoagulability increased thrombotic risk, which created a double hit to flap perfusion. Wei et al. (29) elucidated that combined systemic and procedural risk factors accounted for 70% of the flap failure cases.

Our study identified five independent preoperative predictors. With 44 outcome events, the events per variable (EPV) ratio was 8.8, which was slightly below the conventional 10–20 EPV heuristic for traditional logistic regression. To proactively mitigate the risk of overfitting and enhance robustness, we employed LASSO regression for variable selection and coefficient shrinkage to handle datasets with a relatively high number of candidate predictors. Furthermore, we conducted rigorous bootstrap internal validation with optimism correction (500 repetitions) to yield more reliable performance estimates for new populations. Notably, the model maintained good discrimination (optimism-corrected AUC: 0.87) and calibration in the training set, and demonstrated consistent performance in the validation cohort (AUC: 0.86). These methodological safeguards supported the model’s stability despite the sample size constraint. However, future external validation in larger multi-center cohorts is warranted to further confirm the model’s generalizability and refine the estimates of predictor effects.

Based on the five risk factors identified in our nomogram, we propose the following risk-stratified management strategies, acknowledging that these recommendations are derived from literature and expert consensus. For patients with preoperative hypoalbuminemia (<35 g/L), we suggest nutritional optimization initiated at least 72 h before surgery, including high-protein enteral nutrition or intravenous albumin, with a target of raising albumin to ≥ 35 g/L (30, 31). In cases of elevated D-dimer (> 0.5 mg/L) without high bleeding risk, perioperative prophylactic low-molecular-weight heparin may be considered, and a hypercoagulability workup is advisable for unexplained persistent elevation (32, 33). For Gustilo IIIB/IIIC fractures or soft tissue defects exceeding 50 cm^2^, we recommend early debridement and flap coverage within 72 h, the use of robust perforator flaps, and broad-spectrum antibiotics (34). When operative time exceeds 6 h, employing dual-surgeon teams can help reduce ischemia time. Additionally, intraoperative perfusion monitoring may be considered if prolonged surgery is anticipated (35).

Our study has several limitations that should be considered when interpreting results. First, its retrospective design resulted in selection bias. Patients with incomplete records were excluded, which may have underestimated risk in severely injured patients. Second, regarding model validation, our validation cohort was generated by a random split of our single-center retrospective dataset. While this provided an essential step of internal validation, it did not constitute a true external validation. Consequently, the evidence for the model’s generalizability to other clinical settings, where patient demographics, surgical practices, and perioperative protocols may differ, remained limited. The model’s good performance in our internal validation cohort was encouraging but should be interpreted within this context. Third, the dichotomization of continuous predictors was performed to enhance the clinical usability of the nomogram. We acknowledge that this process may lead to a loss of information and potentially reduce the model’s discriminative power. The choice of thresholds was grounded in existing literature. However, due to the moderate sample size and limited number of events (*n* = 44) in our single-center cohort, we did not perform restricted cubic spline (RCS) analysis to confirm whether the selected cutoffs such as 6 h and 50 cm^2^, represented true risk inflection points. Therefore, these thresholds should be interpreted as clinically practical rather than statistically derived, and they may limit generalizability to populations where different cutoffs are clinically relevant. Fourth, the interaction analysis between D-dimer and albumin was performed as a *post-hoc* exploratory analysis; therefore, the observed synergistic effect should be interpreted as hypothesis-generating and requires confirmation in independent cohorts. Finally, we did not evaluate long-term outcomes. Future studies should address these gaps with prospective, multi-center designs and, if sample size permits, employ RCS analysis to identify optimal cutoffs for continuous predictors.

## Conclusion

Our study identified five independent preoperative risk factors for free flap necrosis in lower limb soft tissue reconstruction: Gustilo-Anderson classification IIIB/IIIC, preoperative D-dimer > 0.5 mg/L, operative time > 6 h, preoperative albumin < 35 g/L, and defect size > 50 cm^2^. Based on these factors, we developed and internally validated a nomogram with robust discrimination, excellent calibration and meaningful clinical utility. However, due to the retrospective, single-center design and reliance on internal validation, the model’s generalizability requires confirmation by prospective multicenter external validation before potential clinical application.

## Data Availability

The raw data supporting the conclusions of this article will be made available by the authors, without undue reservation.
